# Diagnostic and prognostic values of serum activin-a levels in patients with acute respiratory distress syndrome

**DOI:** 10.1186/s12890-019-0879-6

**Published:** 2019-06-25

**Authors:** Jee-min Kim, Jung-Kyu Lee, Sun Mi Choi, Jinwoo Lee, Young Sik Park, Chang-Hoon Lee, Jae-Joon Yim, Chul-Gyu Yoo, Young Whan Kim, Sung Koo Han, Sang-Min Lee

**Affiliations:** 1 Division of Pulmonary and Critical Care Medicine, Department of Internal Medicine, National Medical Center, 245 Eulji-ro, Joong-gu, Seoul, 04564 Republic of Korea; 2 Division of Pulmonary and Critical Care Medicine, Seoul Metropolitan Government-Seoul National University Boramae Medical Center, 425 Sindaebang dong, Dongjak-gu, Seoul, 07061 Republic of Korea; 3 Department of Internal Medicine, Seoul National University College of Medicine, 103 Daehak-ro, Seoul, 03080 Republic of Korea; 4 Division of Pulmonary and Critical Care Medicine, Department of Internal Medicine, Seoul National University College of Medicine, Seoul National University Hospital, 101 Daehak-ro, Jongno-gu, Seoul, 03080 Republic of Korea

**Keywords:** Activins, Hospital mortality, Intensive care units, Prognosis, Respiratory distress syndrome, adult

## Abstract

**Background:**

We aimed to evaluate whether serum activin-A levels are elevated and have any value in predicting severity and prognosis in acute respiratory distress syndrome (ARDS).

**Methods:**

Retrospective cohort study was performed with patients who were admitted to MICU with diagnosis of ARDS and have serum samples stored within 48 h of Intensive care unit (ICU) admission between March 2013 and December 2016 at a single tertiary referral hospital. Serum activin-A levels were measured with ELISA kit, and were compared with those of normal healthy control and non-ARDS sepsis patients.

**Results:**

Total 97 ARDS patients were included for the study. Levels of Activin-A were elevated in ARDS patients compared to those of healthy controls (Log-transformed activin-A levels 2.89 ± 0.36 vs. 2.34 ± 0.11, *p* < 0.001, absolute activin-A levels 1525.6 ± 1060.98 vs. 225.9 ± 30.1, *p* = 0.016) and non-ARDS sepsis patients (Log-transformed activin-A levels 2.89 ± 0.36 vs. 2.73 ± 0.34, *p* = 0.002, Absolute activin-A levels 1525.6 ± 1060.98 vs. 754.8 ± 123.5 pg/mL, *p* = 0.036). When excluding five outliers with extremely high activin-A levels, activin-A showed statistically significant correlation with in-hospital mortalities (In-hospital survivors 676.2 ± 407 vs. non-survivors 897.9 ± 561.9 pg/mL, *p* = 0.047). In predicting in-hospital mortality, serum activin-A concentrations showed superior area under curve compared to that of Acute physiologic and chronic health evaluation II scores (0.653; 95% CI [0541, 0.765] vs. 0.591, 95% CI [0.471, 0.710]). With cut-off level of 708 pg/mL, those with high serum activin-A levels had more than twofold increased risk of in-hospital mortalities. However, those relations were missing when outliers were in.

**Conclusions:**

Serum activin-A levels in ARDS patients are elevated. However, its levels are weakly associated with ARDS outcomes.

**Electronic supplementary material:**

The online version of this article (10.1186/s12890-019-0879-6) contains supplementary material, which is available to authorized users.

## Introduction

Acute Respiratory Distress Syndrome (ARDS), characterized by diffuse inflammation and increased pulmonary vascular permeability with widespread fibrosis later on, continues to be a major healthcare burden with a mortality of 27–45% depending on reports [1]. Several scoring systems have been proposed for early identification of patients at risk of developing ARDS and prediction of survival in ARDS patients. Those tools include Lung Injury Prediction Score (LIPS), Early Acute Lung Injury (EALI) score, and Surgical Lung Injury Prediction (SLIP), but their use is limited in real clinical world for they have low positive predictive value and some of these scoring systems are only for pre-operative patients [2–4]. For evaluating severity and predicting prognosis of ARDS patients, Acute Physiology and Chronic Health Evaluation (APACHE II) and Sequential Organ Failure Assessment (SOFA) scores are widely used, but these instruments have shortcomings in that they are complex, time-consuming to calculate, and they are not specifically designed for ARDS patients [5, 6].

For these reasons, there have been increasing needs for biomarker which could reflect severity or prognosis of ARDS patients. Serum activin-A, a member of transforming growth factor (TGF-ß) superfamily, is a pleiotrophic regulator of cell development and function and its level is elevated in both acute and chronic inflammation [7, 8]. It has already been studied that serum activin-A has a prognostic role in sepsis [9]. Furthermore, there are increasing evidence that activin-A plays an important role in several lung diseases including ARDS. In murine model, selective overexpression of activin-A in airway caused pulmonary pathology which was similar to acute lung injury(ALI)/ARDS. In human, it has been reported that patients with ARDS have increased level of activin-A levels in broncho-alveolar lavage(BAL) fluid [10]. However, levels of activin-A in serum, which is more convenient to measure than performing BAL in clinical practice, in ARDS patients has not been elucidated. Moreover, its relationship with ARDS severity or prognosis is rarely known. We aimed to study whether serum activin-A concentration has any diagnostic or prognostic value for critically ill patients with ARDS.

## Materials and methods

### Study design and participants

We performed a retrospective cohort study with patients who were admitted to medical intensive care unit (ICU) and have serum samples stored within 48 h of ICU admission between March 2013 and December 2016 in Seoul National University Hospital. Among those patients, we retrospectively enrolled patients who were diagnosed with ARDS at the time of ICU admission. ARDS was defined according to the 2012 Berlin definition [11]. To evaluate whether serum activin-A levels are elevated among ARDS patients compared to others, we set two control groups. First is healthy control group consisting of those who visited healthcare center for routine health screening examinations and did not have any specific diseases. The second group consists of critically ill patients without ARDS, who were 1:1 propensity-matched with ARDS patients for age, sex, and APACHE II score. Those were among patients with sepsis who were admitted for ICU within the same period but those who did not have ARDS. All biospecimen for this study was provided by Seoul National University Hospital Human Biobank, a member of National Biobank of Korea, which is supported by the Ministry of Health and Welfare. All samples derived from National Biobank of Korea were obtained with informed consent under institutional review board-approved protocols. (IRB number H-1004-037-315) This study was performed in accordance with the tenets of the Declaration of Helsinki, and this study protocol was approved by the ethics board of Seoul National University Hospital. (IRB number H-1703-166-841).

Severity scores including APACHE II, SOFA, and Simplified Acute Physiologic Score (SAPS II) scores were calculated for each patient. Parameters including arterial oxygen tension (PaO2)/fractional inspired oxygen (FiO2) ratio and laboratory data including C-reactive protein were reviewed for the nearest time of measurement of serum activin-A level. Causes of ARDS, ICU mortality and hospital mortality were reviewed.

### Measurement of serum activin-a concentrations

Measurement of serum activin-A levels was performed in same method as we previously published [9]. Right after the collection, samples were centrifuged for 10 min at 2500 rpm/min and stored at − 80 °C. They were thawed right before the assays. An enzyme-linked immunosorbent assay (Quantikine Human/Mouse/Rat Activin-A Immunoassay;R&D Systems, Abingdon, UK) was performed to measure the activin-A concentration. To confirm intra-assay precision, all samples were assayed in duplicate. The intra-assay coefficients of variation for activin-A was 4.2%, and the lower level of detection was 56.1 pg/mL.

### Outcomes and statistical analysis

The primary outcomes were ICU and hospital mortalities. The secondary outcomes were severity of ARDS, P/F ratio, APACHE II score, SAPS II score, and SOFA score.

The data are presented as mean (±standard deviation) or median (interquartile range) for continuous variables and as number (percentage) for categorical variables. The Mann-Whitney *U* test was used to compare characteristics of survivors and non-survivors. The association between mortality rates and levels of serum activin-A levels were evaluated using Cox proportional hazard models. For the comparison of serum activin-A levels between ARDS patients and control groups, 1:1 propensity score matched Cox proportional hazard analysis was adopted. A receiver-operating characteristic (ROC) curve and multivariate logistic regression were adopted to evaluate the prediction power of ICU/Hospital mortality. Correlations between serum activin-A levels and other prognostic, physiological, and biochemical parameters were analyzed by logistic regression. Odd ratios (OR) and adjusted OR (aOR) were presented with 95% confidence intervals (CI). *P* value less than 0.05 was determined to have statistical significance. SPSS version. 23 (SPSS Inc., Chicago, IL, USA) was used for statistical analysis.

## Results

### Baseline characteristics and clinical features of ARDS patients

Among 577 patients who were admitted to medical ICU and had serum samples stored within 48 h of admission from May 2013 to December 2016, 97 patients were diagnosed with ARDS at the time of ICU admission and were eligible for inclusion (See Additional file 1: Figure S1). Baseline characteristics and clinical features of ARDS of the study population are presented in Table 1. Median age of study population was 67.2, and 64.3% were male. About 17.4% had underlying lung cancer and 52% had underlying malignancies. Ninety-one patients (92.9%) received mechanical ventilation, while 8 patients survived through ARDS with non-invasive ventilation such as high flow nasal cannulas. When defining hypoxemia of PaO2 < 60 mmHg regardless of FiO2 as type 1 respiratory failure and PaCO2 > 45 mmHg with respiratory acidosis as type 2 respiratory failure, most of the patients received mechanical ventilation for type 1 respiratory failure. 85.7% of patients had direct lung injury for the cause of ARDS, and majorities of direct lung injury were pneumonia. 14.3% of patients had indirect lung injury and most of them were related to sepsis. About 67.3% of patients had severe ARDS; their P/F ratio at the time of ICU admission was less than 100. Their mean APACHE II, SOFA, SAPS II score were 23.9, 9.2 and 37.8, respectively (Table 1).

**Table 1:** Baseline demographics and clinical features of ARDS patients

Variables	Total patients (*n* = 97)
Age, years	67.2 (64.3–70.1)
Sex, Male, N(%)	63 (64.3%)
BMI, kg/m^2^	22.5 (21.7–23.4)
Underlying lung disease, N(%)	
Lung cancer	17 (17.4%)
Chronic obstructive lung disease	7 (7.1%)
Idiopathic pulmonary fibrosis	7 (7.1%)
Tuberculosis-destroyed lung	4 (4.1%)
Metastatic cancer to lung	5 (5.1%)
Others	15 (15.3%)
Comorbidities, N(%)	
Hypertension	39 (39.8%)
Diabetes mellitus	25 (25.5%)
Cardiovascular disease	32 (32.7%)
Cerebrovascular disease	10 (10.2%)
Malignancy	51 (52.0%)
Liver cirrhosis	9 (9.2%)
Chronic kidney disease	18 (18.4%)
Intubation, N(%)	91 (92.9%)
Indication for intubation, N(%)	
Type 1 respiratory failure	83 (84.7%)
Type 2 respiratory failure	8 (8.2%)
Cause of ARDS, N(%)	
Direct lung injury	86 (87.8%)
Indirect lung injury	11 (12.2%)
Severity of ARDS	
Mild	3 (3.1%)
Moderate	29 (29.6%)
Severe	65 (67.3%)
APACHE II score	23.9 (21.7–26.1)
SOFA score	9.2 (8.2–10.3)
SAPS II score	37.8 (33.6–42.1)
Mechanical ventilation setting^b^	
PaO2/FiO2 ratio	82 (64–114)
Peak inspiratory pressure, cmH20	25 (21–29)
Tidal volume, mL	440 (398–542)
Minute volume, L	10.0 (9.0–12.0)
Positive end-expiratory pressure, cmH20	7 (5–10)
Serum Activin-A concentration (pg/mL)	1525.62 (472.60–2578.64)
Serum Activin-A concentration^a^	2.89 (2.82–2.96)

*Abbreviations*: *BMI* body mass index, *ARDS* acute respiratory distress syndrome, *APACHE* acute physiologic and chronic health evaluation, *SOFA* sepsis-related organ failure, *SAPS* simplified acute physiologic score, *LIPS* lung injury prediction score, *FiO2* fraction of inspired oxygen, *PaO2* partial pressure arterial oxygen

^a^log transformation of levels of serum activin-A was done

^b^values are presented as median(interquartile range)

### Clinical course of ARDS patients

66.3% of patients received inotropics at some point of ICU care, and about half the patients (46.9%) received nitric oxide in addition to mechanical ventilation. 81.6% of patients received corticosteroids for some reasons; ARDS, sepsis, Pneumocystic jiverocii pneumonia, acute exacerbation of idiopathic pulmonary fibrosis, etc. their average durations of mechanical ventilation were 25 days. Successful extubation was done in 41 patients (41.8%), and 26 patients (26.5%) ended up receiving tracheostomy. Median length of ICU and hospital stays was 11 and 26 days, respectively. 58.2% of patients died in ICU, and 64.3% of patients died in hospital (Table 2).

**Table 2:** Clinical course of ARDS patients

Variables	Total patients (*n* = 97)
Use of adjunctive therapies, N(%)	
Inotropics	65 (66.3%)
Corticosteroid	80 (81.6%)
Nitric Oxide	46 (46.9%)
Prone position	17 (17.3%)
Continuous renal replacement therapy	21 (21.4%)
Extracorporeal membrane oxygenation	6 (6.1%)
Duration of mechanical ventilation	10 (5–20)
Extubation, N(%)	41 (41.8%)
Tracheostomy, N(%)	26 (26.5%)
ICU length of stay, days	11 (6–17)
Hospital length of stay, days	26 (14–50)
ICU mortality, N(%)	57 (58.2%)
Overall In-hospital mortality	63 (64.3%)
30-day in-hospital mortality	45 (45.9%)
60-day in-hospital mortality	60 (61.2%)

*Abbreviations*: *ICU* intensive care units

Continuous variables are presented as median(interquartile range)

### Levels of serum activin-a in ARDS patients

**Fig. 1:**
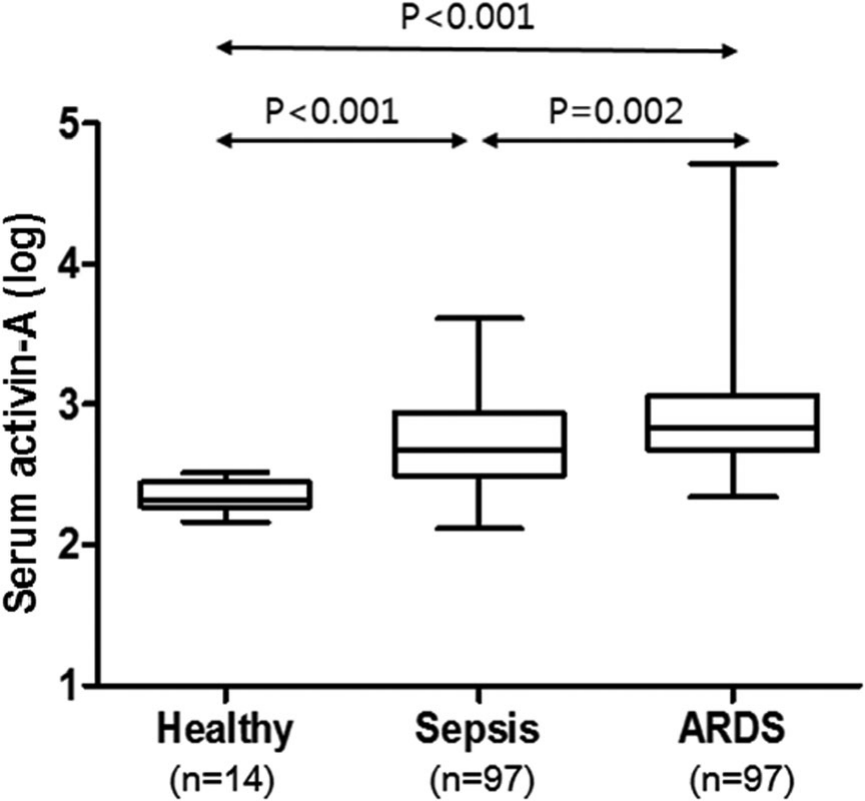
Comparison of concentration of serum activin-A among healthy controls, critically-ill patients with sepsis, and patients with ARDS. Serum activin-A levels are log transformed. Healthy controls are age and sex-matched with ARDS patients. Those with sepsis patients were age, sex, and APACHE-II score matched with those of ARDS patients

Average concentration of activin-A in ARDS patients was 1525.62 pg/mL, ranging from 219 to 51,412 pg/mL. Due to its wide distribution, log transformation was done to compare its value with control groups. To evaluate whether serum activin-A level is elevated in ARDS patients, we measured serum activin-A levels of 14 healthy controls who were age and sex-matched with ARDS patients. Compared to healthy control, serum levels of activin-A were significantly elevated in ARDS (2.89 ± 0.36 vs. 2.34 ± 0.11, *p* < 0.001, absolute activin-A levels 1525.6 ± 1060.98 vs. 225.9 ± 30.1 pg/mL, *p* = 0.016) (Fig. 1). For confirming whether serum activin-A levels are elevated in ARDS patients compared to non-ARDS ICU patients, we selected age, sex, and APACHE II score-matched ICU patients who were admitted to ICU for sepsis without ARDS. Of propensity score-matched 97 patients with sepsis, there were no difference in age, sex, and APACHE II score compared to those of ARDS patients. Patients with ARDS had serum levels of activin-A that were higher than those of non-ARDS sepsis patients, which was statistically significant (2.89 ± 0.36 vs. 2.73 ± 0.34, *p* = 0.002, Absolute activin-A levels 1525.6 ± 1060.98 vs. 754.8 ± 123.5 pg/mL, *p* = 0.036) (Fig. 1). However, within ARDS patients there were no statistically significant differences in serum activin-A levels between septic ARDS and non-septic ARDS patients (2.92 ± 0.31 vs. 2.88 ± 0.07, *p* = 0.738). Furthermore, there were no differences in serum activin-A levels between direct ARDS and indirect ARDS (2.90 ± 0.79 vs. 2.81 ± 0.18, *p* = 0.776, Absolute activin-A levels 1577.2 ± 1082 vs. 1077.2 ± 314.3, *p* = 0.441) (See Additional file 1: Figure S3).

### Discriminant power of serum activin-a levels in ARDS patients and its association with other clinical variables

There were no differences in serum activin-A levels between mild to moderate and severe ARDS patients. (899.4 ± 399.3 vs. 1833.9 ± 1569.6 pg/mL, *p* = 0.410). There was no significant correlation between serum activin-A levels and P/F ratio at ICU admission (See Additional file 1: Figure S2). When evaluating association between serum activin-A levels with other variables that are known to reflect severity of disease, serum activin-A levels showed statistically significant association between SAPS II score, but showed no significant association with APACHE II, SOFA score, or level of C-reactive protein (See Additional file 1: Table S1).

### Prediction of clinical outcomes with serum activin-a levels

**Fig. 2:**
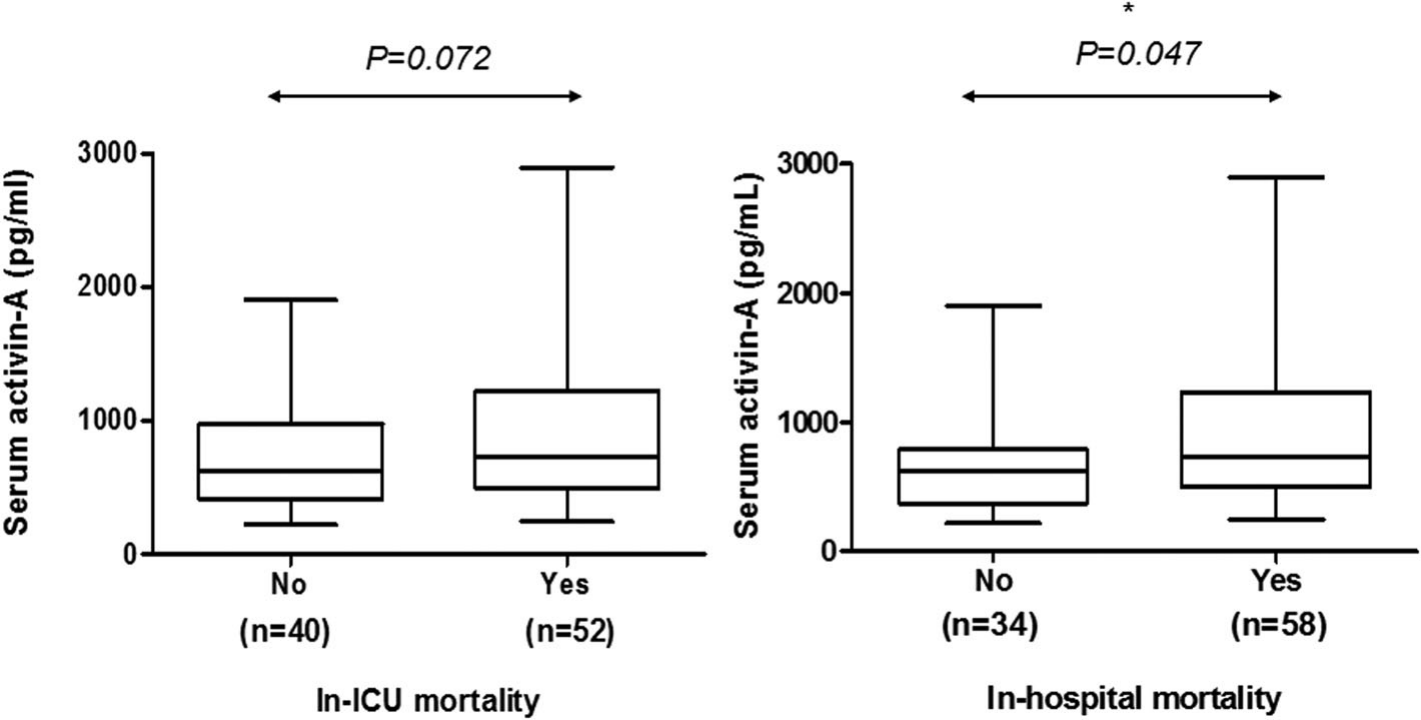
Serum activin-A concentration according to hospital outcomes. (Analysis were performed excluding 5 outliers)

Serum activin-A levels were compared between survivors and non-survivors. Those who died during ICU stay or after ICU stay within hospitalization tended to have higher serum activin-A levels compared to those who survived, but there were no statistical significance. Because some patients showed extremely high serum activin-A levels (> 3000 pg/mL), we performed another analysis after excluding those outliers (all those 5 patients with extremely high serum activin-A levels were ICU/Hospital non-survivors). When excluding these outliers, there was significant difference in serum activin-A levels between survivors and non-survivors during hospitalization (Fig. 2) (See Additional file 1: Table S2). When dividing patients into 4 quartiles according to their serum activin-A levels, Quartile 4 showed highest in-hospital mortality and shortest median survival days while Quartile 1 showed lowest in-hospital mortality and longest median survival days (See Additional file 1: Table S3).

**Fig. 3:**
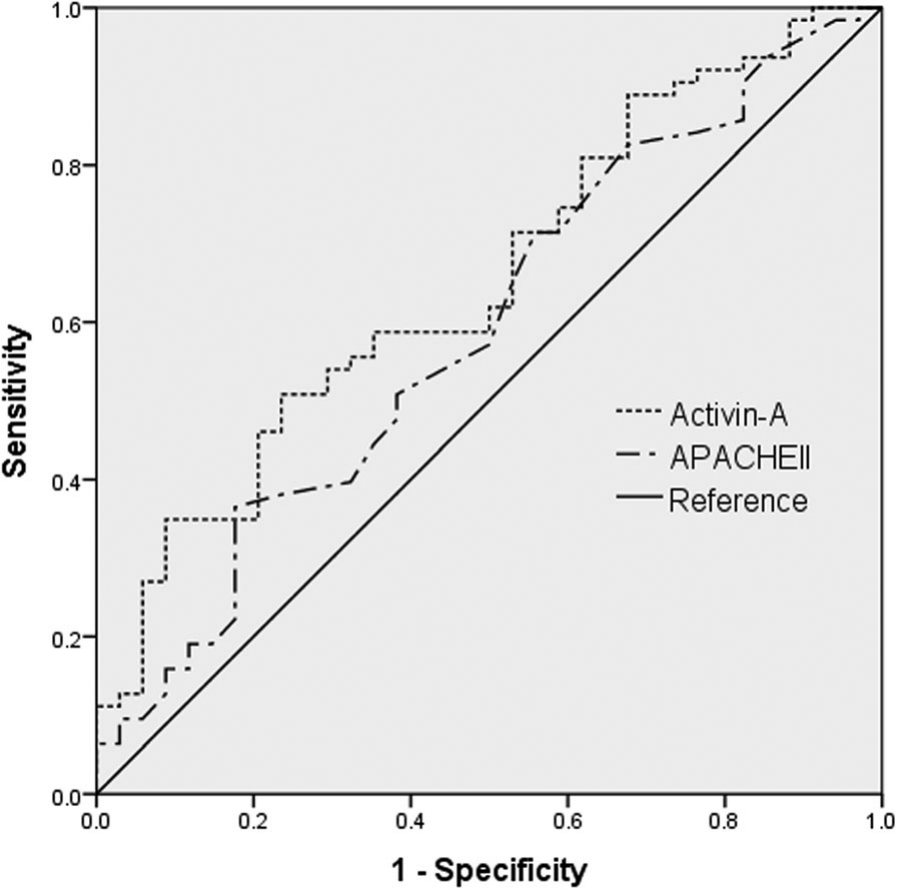
Receiver-operating characteristic curves for predicting in-hospital mortality. Serum activin-A level is presented as dotted line, APACHE II score as chain line, and reference value as solid line

A ROC curve was used to evaluate whether the serum activin-A levels could be useful to predict in-hospital mortality. Predicting the mortality in the study population, the area under the ROC curve (AUC) was calculated as 0.635 (95% CI: 0.252, 0.745, *p* = 0.024). This was higher than that of APACHE II score, of which AUC was calculated as 0.548 (95% CI: 0.432, 0.665, *p* = 0.418) (Table 3, Fig. 3). The cut-off level of 708 pg/mL for serum activin-A concentration had a sensitivity of 55.8% and specificity of 67.2%. In patients with serum activin-A levels of 708 pg/mL or higher, the risk of in-hospital mortality was 2.61-fold higher than in those with serum activin-A levels less than 708 pg/mL in the univariate analysis. This result was consistent in the multivariate analysis after the adjustment with age, sex, SOFA, SAPS II, and APACHE II. (aOR 2.64, 95% CI: 1.04–6.70, *p* = 0.041) (Table 4). However, when including those 5 outliers, none of the statistical significance regarding serum activin-A levels and ARDS outcomes was maintained.

**Table 3:** ROC curve of serum activin-A, APACHE II score in predicting in-hospital, ICU mortality^a^

Parameter	In-hospital mortality			ICU mortality		
	AUC	95% CI	*p*-value	AUC	95% CI	*p*-value
Activin-A	0.653	0.541, 0.765	0.013	0.635	0.525, 0.745	0.024
APACHE II	0.591	0.471, 0.710	0.142	0.548	0.432, 0.665	0.418

*Abbreviations*: *ICU* intensive care units, *APACHE* acute physiologic and chronic health evaluation

^a^*N* = 92 (5 Outliers are excluded)

**Table 4:** ^a^Serum activin-A level in predicting in-hospital mortality

Variables	Univariate		Multivariate^b^	
	Unadjusted OR	*P* value	Adjusted OR	*P* value
Prognostic values				
APACHE II score	1.03 (0.99, 1.08)	0.147	1.05 (0.99, 1.10)	0.061
SOFA score	1.03 (0.95, 1.12)	0.443	1.06 (0.97, 1.16)	0.186
SAPS II score	1.02 (0.99, 1.04)	0.103	1.01 (0.99, 1.04)	0.278
High serum activin-A (> 708 pg/mL)	2.61 (1.09, 6.26)	0.031	2.64 (1.04, 6.70)	0.041

*Abbreviations*: *APACHE* acute physiologic and chronic health evaluation, *SOFA* Sepsis-related organ failure, *SAPS* simplified acute physiologic score

^a^*N* = 92 (5 Outliers are excluded)

^b^Adjusted by APACHE II, SOFA, SAPS II score, and serum activin-A concentrations

## Discussion

In this study, we found that serum activin-A levels are elevated in ARDS, but its levels are weakly associated with hospital mortalities.

There are few biomarkers that have been proposed to reflect early identification and predicting prognosis of ARDS. In one study, plasma concentration of angiopoietin-2 in combination with LIPS score improved predictability of ARDS than using LIPS score alone [12, 13]. In another study, lower uric acid was associated with lower in-hospital mortality with a cut-off level of uric acid as 3.0 mg/dL [14]. However evidence regarding these biomarkers is scarce so they are not in widespread use in clinical field. Activin-A is a member of transforming growth factor-ß superfamily and is a pleiotropic regulator of cell development and function [7]. It is known to be involved in both proinflammatory, antiinflammtory reaction, and tissue-remodeling activities [15, 16]. Increasing evidences have shown that activin-A plays an important role in various pulmonary diseases, such as asthma and chronic obstructive lung disease [8, 17]. Role of activin-A in ARDS has been proposed in murine models. Apostolous et al reported that overexpression of activin-A in mouse airways caused pulmonary pathology reminiscent of acute lung injury. Furthermore, broncho-alveolar lavage fluid of ARDS patients showed high activin-A levels compared to those in non-ARDS controls [10]. However, little is known about the change of serum activin-A levels in ARDS patients.

Compared to both healthy controls and critically ill patients with sepsis, patients with ARDS had a significantly higher levels of serum activin-A. Although there were no differences of serum activin-A levels according to ARDS severity, those with higher serum activin-A levels, when excluding 5 outliers with extremely high activin-A levels, showed higher in-hospital mortality. Cut-off value of serum activin-A 708 pg/mL or higher was associated with more than twofold increase in in-hospital mortality. Although serum activin-A concentration was not correlated with ARDS severity, those with activin-A levels higher than 708 pg/mL had significantly lower P/F ratio which implies more severe disease than those with lower activin-A levels (See Additional file 1: Table S4). However, correlations disappeared when including the 5 outliers.

There are few limitations in this study. Of all, this is single-center retrospective study conducted in single unit of medical ICU. There are some differences in patient characteristics from known ARDS patients from recent large epidemiologic study of ARDS patients. Compared to the study of ARDS in 2016, more patients in this study were consisted of severe ARDS and in-hospital mortalities were higher [18]. More than half of patients had underlying malignancies. The hospital where the study was conducted is a tertiary referral hospital and large portion of the patients are immunocompromised with hemato-oncologic diseases, which could have probably led to more severe ARDS. Nevertheless, we found out that there was no statistically significant difference in serum activin-A concentrations between patients with and without cancers. Furthermore, almost 80% of the patients received corticosteroids. Thirty-three patients (34%) received corticosteroids for ARDS itself. Although steroid use is not recommended for treatment of ARDS, We consider this data reflects how widespread steroids have been used in ARDS in real world practice to some extent. Nevertheless, there were no statistically significant differences in serum activin-A levels between patients with and without steroid treatment. Another limitation is that there is lack of data regarding how well low tidal ventilation and optimal PEEP titration were performed during the study period. Although there is usual in-hospital practice guideline regarding lung protective ventilation for ARDS patients, there is no available date to evaluate how well the clinicians adhered to the recommendations, which could have affected the patient outcomes. Another limitation is that because the blood samples were taken during 48 h of ICU admission, there could have been a time lag as long as 2 days between ICU admission and blood samples. This could have exerted influence on relationship between serum activin-A levels and the patients’ outcomes. Moreover, we did not measure sequential levels of activin-A. In order to further elucidate the prognostic role of activin-A, comparing serial changes in activin-A between survivors and non-survivors would be helpful. In addition to acute inflammation, activin-A is known to play a role in late phase of inflammation and airway remodeling [19, 20]. Thus, it would be interesting to see whether serum activin-A increases in late fibrotic phase in ARDS and if its levels are modulated by administrating anti-inflammatory drugs such as corticosteroids. Another limitation of this study is that we could not find proper explanation for patients with extremely high serum activin-A concentrations. Of 97 patients, 5 patients showed very high serum activin-A concentrations; that is, activin-A over 3000 pg/mL. All of them had ARDS after direct lung injuries. Four patients had severe ARDS and 1 patient had mild to moderate ARDS at the time of ICU admission. All of them did not survive through ARDS and died in ICU. We tried to find out shared factors among these patients which could contribute to extremely high serum activin-A levels but could not find any. Future studies are needed to explain these unusually high levels of serum activin-A levels. Lastly, unlike many studies that ARDS after direct lung injury had unfavorable prognosis compared to indirect lung injury, there were no differences in serum activin-A levels or in-hospital mortalities between two groups in this study [21–23]. Because this study was performed in medical ICU, majority of patients had direct lung injury-related ARDS. ARDS patients with indirect lung injuries, for example post-op ARDS, transfusion-related ARDS, trauma-related ARDS, could have been missed for those with indirect lung injuries are generally admitted to surgical ICU. Further studies are needed to compare serum activin-A levels between direct and indirect lung injuries.

## Conclusions

In conclusion, serum activin-A concentrations are elevated in ARDS, but its levels are weakly associated with ARDS outcomes. Further studies are required to evaluate the practicability and clinical benefit of this biomarker.

## Additional file

Additional file 1: **Table S1.** Association between other variables with serum activin-A. **Table S2.** Prognostic value of serum activin level. **Table S3.** ^a^Difference in survival during hospitalization among Quartiles of serum activin level. **Table S4.** Comparison of patients with low and high serum activin-A with cut-off value 708pg/mL. **Figure S1.** Flow diagram of the study population. **Figure S2.** Serum activin-A concentration according to ARDS severity and PaO2/FiO2. **Figure S3.** Serum activin-A concentration between indirect and direct ARDS patients. **Figure S4.** Comparisons of serum activin-A concentrations among healthy control, patients with sepsis (without ARDS), and patients with direct lung injury. (DOCX 186 kb)

## Data Availability

The datasets used and/or analyzed during the preset study are available from the corresponding author on reasonable request.

## Abbreviations

APACHE: Acute Physiologic and Chronic Health Evaluation
ARDS: Acute Respiratory Distress Syndrome
BMI: Body Mass Index
ELISA: Enzyme-Linked ImmunoSorbent Assay
FiO2: Fraction of Inspired Oxygen
ICU: Intensive Care Units
IPF: Idiopathic Pulmonary Fibrosis
LIPS: Lung Injury Prediction Score
P/F ratio: Partial Pressure Arterial Oxygen/Fraction of Inspired Oxygen
PaO2: Partial Pressure Arterial Oxygen
SAPS: Simplified Acute Physiologic Score
SOFA: Sepsis-related Organ Failure

## References

[1] Burnham EL, Janssen WJ, Riches DW, Moss M, Downey GP (2014). The fibroproliferative response in acute respiratory distress syndrome: mechanisms and clinical significance. Eur Respir J.

[2] Gajic O, Dabbagh O, Park PK, Adesanya A, Chang SY, Hou P, Anderson H, Hoth JJ, Mikkelsen ME, Gentile NT, Gong MN, Talmor D, Bajwa E, Watkins TR, Festic E, Yilmaz M, Iscimen R, Kaufman DA, Esper AM, Sadikot R, Douglas I, Sevransky J, Malinchoc M (2011). Early identification of patients at risk of acute lung injury: evaluation of lung injury prediction score in a multicenter cohort study. Am J Respir Crit Care Med.

[3] Levitt JE, Calfee CS, Goldstein BA, Vojnik R, Matthay MA (2013). Early acute lung injury: criteria for identifying lung injury prior to the need for positive pressure ventilation. Crit Care Med.

[4] Kor DJ, Warner DO, Alsara A, Fernández-Pérez ER, Malinchoc M, Kashyap R, Li G, Gajic O (2011). Derivation and diagnostic accuracy of the surgical lung injury prediction model. Anesthesiology.

[5] Knaus WA, Draper EA, Wagner DP, Zimmerman JE (1985). APACHE II: a severity of disease classification system. Crit Care Med.

[6] Vincent JL, Moreno R, Takala J, Willatts S, De Mendonca A, Bruining H, Reinhart CK, Suter PM, Thijs LG (1996). The SOFA (sepsis-related organ failure assessment) score to describe organ dysfunction/failure. On behalf of the working group on sepsis-related problems of the european society of intensive care medicine. Intensive Care Med.

[7] Hedger M, De Kretser D (2013). The activins and their binding protein, follistatin—diagnostic and therapeutic targets in inflammatory disease and fibrosis. Cytokine Growth Factor Rev.

[8] Verhamme FM, Bracke KR, Amatngalim GD, Verleden GM, Van Pottelberge GR, Hiemstra PS, Joos GF, Brusselle GG (2014). Role of activin-a in cigarette smoke-induced inflammation and COPD. Eur Respir J.

[9] Lee JK, Choi SM, Lee J, Park YS, Lee CH, Yim JJ, Yoo CG, Kim YW, Han SK, Lee SM (2016). Serum activin-a as a predictive and prognostic marker in critically ill patients with sepsis. Respirology.

[10] Apostolou E, Stavropoulos A, Sountoulidis A, Xirakia C, Giaglis S, Protopapadakis E, Ritis K, Mentzelopoulos S, Pasternack A, Foster M, Ritvos O, Tzelepis GE, Andreakos E, Sideras P (2012). Activin-a overexpression in the murine lung causes pathology that simulates acute respiratory distress syndrome. Am J Respir Crit Care Med.

[11] Force ADT (2012). Acute respiratory distress syndrome. JAMA.

[12] Agrawal A, Matthay MA, Kangelaris KN, Stein J, Chu JC, Imp BM, Cortez A, Abbott J, Liu KD, Calfee CS (2013). Plasma angiopoietin-2 predicts the onset of acute lung injury in critically ill patients. Am J Respir Crit Care Med.

[13] Yadav H, Thompson BT, Gajic O (2017). Fifty years of research in ARDS. Is acute respiratory distress syndrome a preventable disease?. Am J Respir Crit Care Med.

[14] Lee HW, Choi SM, Lee J, Park YS, Lee C-H, Yim J-J, Yoo CG, Kim YW, Han SK, Lee SM. Serum uric acid level as a prognostic marker in patients with acute respiratory distress syndrome. J Intensive Med. 2017; doi :0885066617698911. 10.1177/0885066617698911 28351229

[15] Dijke Peter ten, Hill Caroline S (2004). New insights into TGF-β–Smad signalling. Trends in Biochemical Sciences.

[16] Huang HM, Chiou HY, Chang JL (2006). Activin a induces erythroid gene expressions and inhibits mitogenic cytokine-mediated K562 colony formation by activating p38 MAPK. J Cell Biochem.

[17] Kariyawasam HH, Pegorier S, Barkans J, Xanthou G, Aizen M, Ying S, Kay AB, Lloyd CM, Robinson DS (2009). Activin and transforming growth factor-β signaling pathways are activated after allergen challenge in mild asthma. J Allergy Clin Immunol.

[18] Bellani G, Laffey JG, Pham T, Fan E, Brochard L, Esteban A, Ranieri M (2016). Epidemiology, patterns of care, and mortality for patients with acute respiratory distress syndrome in intensive care units in 50 countries. JAMA.

[19] Matsuse T, Ikegami A, Ohga E, Hosoi T, Oka T, Kida K, Fukayama M, Inoue S, Nagase T, Ouchi Y, Fukuchi Y (1996). Expression of immunoreactive activin a protein in remodeling lesions associated with interstitial pulmonary fibrosis. Am J Pathol.

[20] Hardy C, Rolland J, O'hehir R (2015). The immunoregulatory and fibrotic roles of activin a in allergic asthma. Clin Exp Allergy.

[21] Monchi M, Bellenfant F, Cariou A, Joly L-M, Thebert D, Laurent I, Dhainaut JF, Brunet F (1998). Early predictive factors of survival in the acute respiratory distress syndrome: a multivariate analysis. Am J Respir Crit Care Med.

[22] Rouby J-J, Puybasset L, Cluzel P, Richecoeur J, Lu Q, Grenier P (2000). Regional distribution of gas and tissue in acute respiratory distress syndrome. II. Physiological correlations and definition of an ARDS severity score. Intensive Care Med.

[23] Shaver CM, Bastarache JA (2014). Clinical and biological heterogeneity in ARDS: direct versus indirect lung injury. Clin Chest Med.

