# Supplementary material for: Diagnostic and prognostic values of serum activin-a levels in patients with acute respiratory distress syndrome
Source: BMC Pulm Med. 2019 Jun 25;19:115. doi: 10.1186/s12890-019-0879-6 (PMC6593589; doi:10.1186/s12890-019-0879-6)
Supplement: Supplementary file 1 — Table S1. Association between other variables with serum activin-A. Table S2. Prognostic value of serum activin level. Table S3. aDifference in survival during hospitalization among Quartiles of serum activin level. Table S4. Comparison of patients with low and high serum activin-A with cut-off value 708pg/mL. Figure S1. Flow diagram of the study population. Figure S2. Serum activin-A concentration according to ARDS severity and PaO2/FiO2. Figure S3. Serum activin-A concentration between indirect and direct ARDS patients. Figure S4. Comparisons of serum activin-A concentrations among healthy control, patients with sepsis (without ARDS), and patients with direct lung injury. (DOCX 186 kb) [file 12890_2019_879_MOESM1_ESM.docx]

**Table S1. Association between other variables with serum activin-A**

| Variables | Univariate | | Multivariate^a^ | |
| --- | --- | --- | --- | --- |
|  | ß±SE | p-value | ß±SE | p-value |
| Prognostic values  APACHE II score  SOFA score  SAPS II score | 93.3 ± 47.9  40.9 ± 101.4  54.2 ± 25.0 | 0.054  0.687  0.033 | 92.9 ± 48.3  -228.1 ± 151.9  92.2 ± 26.7 | 0.057  0.137  0.001 |
| PaO2/FiO2 ratio, mmHg | -10.0 ± 13.3 | 0.450 | -6.87 ± 13.5 | 0.612 |
| Serum C-reactive protein | 30.9 ± 53.7 | 0.437 | 30.9 ± 53.9 | 0.567 |
| Serum albumin | -178.9 ± 711.5 | 0.802 | -62.9 ± 720.5 | 0.931 |

Abbreviations : APACHE, acute physiologic and chronic health evaluation; SOFA, Sepsis-related organ failure; SAPS, simplified acute physiologic score; FiO2, Fraction of inspired oxygen; PaO2, patial pressure arterial oxygen.

^a^Adjusted by age, sex, APACHE II score

**Table S2. Prognostic value of serum activin level**

| **Total patients** |  |  |  |
| --- | --- | --- | --- |
| **ICU mortality** | **No (n=40)** | **Yes (n=57)** | **p-value** |
| Serum activin (pg/mL) | 705.0 | 2010.5 | 0.197 |
| **In-hospital mortality** | **No (n=34)** | **Yes (n=63)** | **P-value** |
| Serum activin (pg/mL) | 676.2 | 1984.1 | 0.241 |

**Excluding outliers**

| **ICU mortality** | **No (n=40)** | **Yes (n=52)** | **p-value** |
| --- | --- | --- | --- |
| Serum activin (pg/mL) | 705.0 | 901.3 | 0.072 |
| **In-hospital mortality** | **No (n=34)** | **Yes (n=58)** | **P-value** |
| Serum activin (pg/mL) | 676.2 | 897.9 | 0.047 |

Abbreviations: ICU; intensive care units

**Table S3. ^a^Difference in survival during hospitalization among Quartiles of serum activin level.**

|  | **Hospital mortality, N(%)** | **Median survival, days** |
| --- | --- | --- |
| **Quartile 1** | 12 (50.0%) | 46 ± 12 |
| **Quartile 2** | 14 (58.3%) | 44 ± 19 |
| **Quartile 3** | 15 (62.5%) | 44 ± 13 |
| **Quartile** | 22 (88.0%) | 23 ± 5 |

^a^Analysis were performed excluding 5 outliers (n=92)

Abbreviations: ICU; intensive care units

**Table S4. Comparison of patients with low and high serum activin-A with cut-off value 708pg/mL**

|  | **Low activin-A**  **(n=51)** | **High activin-A**  **(n=46)** | **P-value** |
| --- | --- | --- | --- |
| **ARDS severity, N(%)**  **Mild to moderate**  **Severe** | 21 (41.2)  30 (58.8) | 11 (23.9)  35 (76.1) | 0.055 |
| **PaO_2_/FiO_2_** | 100.7±44.9 | 84.8±33.6 | 0.043 |
| **APACHE II** | 22±11 | 25±9 | 0.223 |
| **SOFA** | 8.1±5.3 | 10.2±5.1 | 0.052 |
| **SAPS II** | 34±19 | 41±21 | 0.109 |
| **In-ICU mortality, N(%)** | 25 (49.0) | 32 (69.6) | 0.062 |
| **In-hospital mortality, N(%)** | 28 (54.9) | 35 (76.1) | 0.035 |
| Abbreviations : ARDS, acute respiratory distress syndrome, APACHE, acute physiologic and chronic health evaluation; SOFA, Sepsis-related organ failure; SAPS, simplified acute physiologic score; FiO2, Fraction of inspired oxygen; PaO2, patial pressure arterial oxygen. ICU, intensive care unit | | | |


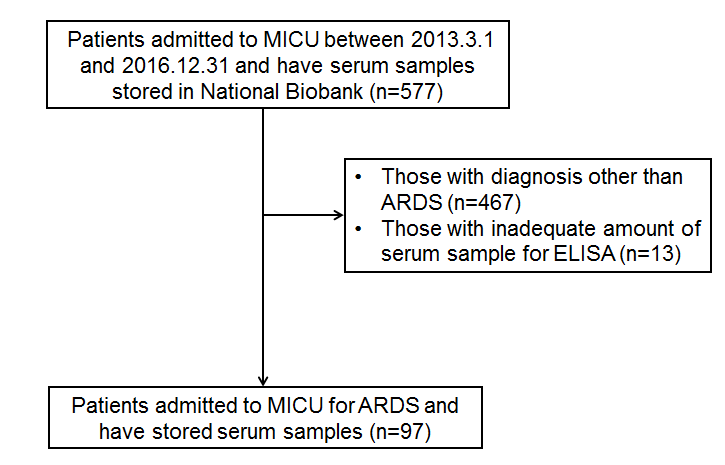


**Figure S1. Flow diagram of the study population**

Abbreviations: MICU, medical intensive care unit; ARDS, acute respiratory distress syndrome; ELISA, enzyme-linked immunosorbent assay

**
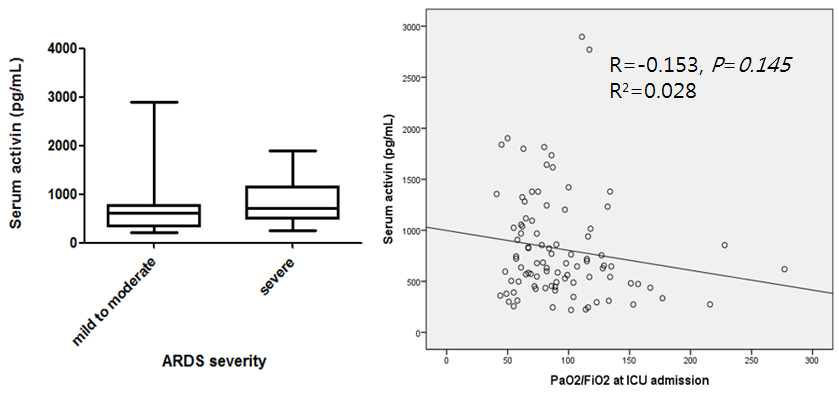
**

**Figure S2. Serum activin-A concentration according to ARDS severity and PaO2/FiO2.**

(a) There are no statistically significant differences in serum activin-A levels between those with mild to moderate ARDS and severe ARDS.

(b) There are no statistically significant differences between serum activin-A levels and PaO2/FiO2 at the time of ICU admission.

Abbreviations : PaO2, patial pressure arterial oxygen; FiO2, fraction of inspired oxygen; ARDS, acute respiratory distress syndrome; ICU, intensive care unit


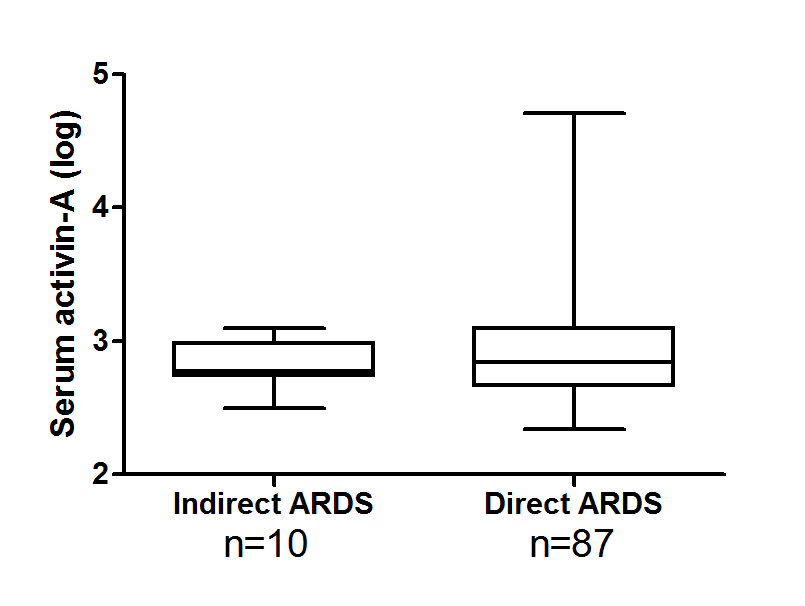


**Figure S3. Serum activin-A concentration between indirect and direct ARDS patients.**

**
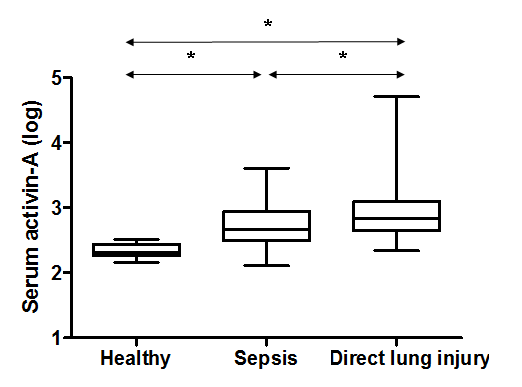
**

**Figure S4. Comparisons of serum activin-A concentrations among healthy control, patients with sepsis (without ARDS), and patients with direct lung injury.**
